# Placebo- and Nocebo-Effects in Cognitive Neuroenhancement: When Expectation Shapes Perception

**DOI:** 10.3389/fpsyt.2019.00498

**Published:** 2019-07-12

**Authors:** Alexander Winkler, Christiane Hermann

**Affiliations:** Department of Clinical Psychology and Psychotherapy, Justus-Liebig-University, Giessen, Germany

**Keywords:** placebo, nocebo, neuroenhancement, expectation, cognitive, performance, drugs

## Abstract

**Objective:** The number of students using prescription drugs to improve cognitive performance has increased within the last years. There is first evidence that the expectation to receive a performance-enhancing drug alone can result in improved perceived and actual cognitive performance, suggesting a substantial placebo effect. In addition, expecting a placebo can result in lower perceived and actual cognitive performance, suggesting a nocebo effect. Yet, the underlying mechanisms of these effects remain to be elucidated. The aim of our study was to investigate whether the expectation of receiving a performance-increasing drug or a performance-impairing drug leads to changes in actual and perceived cognitive performance, compared to a control group without expectation manipulation.

**Methods:** A total of *N* = 75 healthy adults were recruited for an experiment to “try cognitive performance-modulating drugs.” A participant’s actual cognitive performance (alertness, working memory, sustained attention, and divided attention) using the standardized test of attentional performance (TAP) as well as their performance expectation were assessed. Participants were randomly assigned in equal numbers to either receiving a placebo performance increasing nasal spray (“Modafinil”) or a nocebo performance impairing nasal spray (“Vividrin^®^”) or no nasal spray (natural history). After placebo/nocebo nasal spray administration, cognitive performance was reassessed. Subsequent to the second assessment, participants rated their perceived change in cognitive performance, as well as adverse symptoms.

**Results:** Unlike hypothesized, a positive or negative performance expectation did not result in changes in actual performance, corresponding to the induced expectation. Participants in the placebo-Modafinil group rated their perceived change in cognitive performance subsequent to the application of the nasal spray significantly better (*d* = 1.16) compared to the nocebo-Vividrin^®^ group. Additionally, participants who expected to receive Modafinil felt less tired than participants in the Vividrin^®^ group (*d* = 0.96).

**Conclusion:** Manipulation of performance expectation affects the perceived change in performance and tiredness, but not the actual cognitive performance in healthy adults. This may explain why college students use such drugs despite their little impact on actual cognitive functioning.

## Introduction

The number of students using prescription drugs to improve cognitive performance without medical indication has increased over the last years, in spite of the potential risks associated with this use (1). Prevalence rates of non-medical stimulant use of 8.3% (lifetime) and 5.9% (past-year) in a sample of 4,580 US college students (2), 4.3% (lifetime) in a representative sample of 1,128 adults in the German population (3), and a lifetime prevalence rate of 6.5% among Australian university students (4) have been reported

Intriguingly, findings about the actual cognitive enhancement effects of stimulants in non-clinical populations are heterogeneous, suggesting a limited benefit at best (5–7). For example, Ilieva et al. (8) demonstrated that, in healthy participants, a dose of mixed-amphetamine salts enhanced the perceived, but not the actual cognitive ability, suggesting that pharmacological neuroenhancement may exclusively boost the subjective perception of cognitive performance. Interestingly, even if actual performance is improved after drug intake, this might at least partially be accounted for by performance expectation (9). Using a balanced placebo design, Cropsey et al. (9) compared the pharmacological versus expectancy effects of mixed amphetamine salts on cognitive performance in college students. Administered amphetamine salts enhanced cognitive performance in only 2 of 31 subtests of a neuropsychological test battery. Expected administration of the stimulant medication yielded improved perceived and actual cognitive performance, regardless of the group allocation (placebo vs. mixed amphetamine salts) (9). Likewise, Dawkins et al. (10) were able to show that expected caffeine intake improved attention regardless of whether students had consumed caffeinated or decaffeinated coffee in a balanced placebo design.

Aside from studies assessing placebo effects using mixed amphetamine salts (8, 9), caffeine (10, 11), or nicotine (12), studies investigating placebo and nocebo effects on cognitive neuroenhancement have either relied on administering placebo pills or used various psychological interventions (e.g., verbal suggestions) in order to manipulate performance expectation.

Among the studies utilizing placebo pills to manipulate performance expectation, only few studies have directly addressed whether placebo administration is effective in inducing cognitive neuroenhancement measured subjectively and/or objectively. Looby and Earleywine (13) showed that the expectation to receive methylphenidate enhances subjective arousal, but neither perceived nor actual cognitive performance. In fact, such an expectation even tended to impair cognitive performance (13). Szemerszky et al. (14) reported a detrimental effect of a placebo pill on perceived performance in a 14-min vigilance task when the pill was given together with information about its (putative) negative cognitive effects (14). However, in this study, actual cognitive performance was not assessed. Furthermore, there was no increase in symptom reports in the nocebo group (14). Notably, only non-specific bodily symptoms (e.g., abdominal pain, headache, itching) were assessed. Moreover, participants were not specifically informed about potentials side effects of the pill, which was described as a mild sedative.

There are two studies suggesting a placebo effect on objective measures of cognitive performance (15, 16). For example, in healthy seniors, a 2-week intake of a placebo pill enhanced memory and attention performance in comparison to a no pill control condition (15). Interestingly, expectancy of improvement and actual improvement of cognitive performance were correlated, though small in magnitude. In two double-blind randomized-controlled experiments among university students, Colagiuri and Boakes (16) were able to demonstrate that participants who believed they had been allocated to the cognitive-enhancing drug group, due to false (positive) feedback given about their cognitive performance, performed better than those who believed they had been given a placebo.

In one of the very few studies manipulating performance expectation without pill administration, Fuhr and Werle (17) found neither an effect of a mental training based on verbal suggestion nor of the information about the effectiveness of the training on actual cognitive performance. In one of the few studies including both a placebo and a nocebo instruction, the expectation that a tone of a specific frequency will improve or impair cognitive performance strongly affected perceived, but not actual cognitive performance (18). Szemerszky et al. (14) demonstrated a negative effect of a sham magnetic field on perceived performance in a 14-min vigilance task. Unfortunately, actual cognitive performance was not assessed. Moreover, no change in symptom reports was noted (14). There are some studies supporting placebo effects on objective measures of cognitive performance (19–23). For example, sham subliminal presentation of the answers in a knowledge test improved the test scores in college students (20). Fluid intelligence was higher subsequent to a working memory training (1 h) in participants expecting an intelligence boost as compared to participants with no expectation regarding the outcome of the training (Foroughi et al., (21). Turi et al. (22) found a cognitive placebo effect on objective performance measures, but no effect on expectation and perceived performance, using a sham non-invasive brain stimulation technique. Colagiuri et al. (19) demonstrated both a placebo and a nocebo effect in a large sample of university students completing an implicit learning task while being exposed to an odor supposedly enhancing or impairing cognitive performance or having no effect at all. Participants given positive information responded faster; participants given negative information responded slower in cued reaction time trials as compared to the control group (19). Turi et al. (23) demonstrated that a sham non-invasive brain stimulation was able to increase (placebo condition) or decrease (nocebo condition) expected and perceived cognitive performance. Placebo and nocebo effects were also manifest in response accuracy in a reward-based learning performance test (23).

In sum, despite the heterogeneity of findings in the current literature, there is first evidence for placebo and nocebo effects on cognitive performance. However, the influence of such placebo and nocebo instructions has been directly compared only in very few studies [e.g., Ref. (18)]. Additionally, the influence of such placebo/nocebo expectations on cognitive performance has not consistently been evaluated both subjectively and objectively. In the present study, we used a randomized controlled parallel group design to evaluate the effects of expecting a performance-increasing drug (placebo) or a performance-impairing drug (nocebo) on change in performance expectation, actual and perceived cognitive performance, and adverse somatic symptoms (“side effects”), compared to a control group without expectation manipulation, in a sample of 75 college students. We hypothesized that participants in the placebo group would show a higher and participants in the nocebo group a lower performance expectation compared to the control group. We also hypothesized that, depending on the positive or negative performance expectation, perceived and actual performance in a standardized test battery of attention measures would be altered in comparison to the control condition. Additionally, we hypothesized that participants will specifically endorse those adverse symptoms that were described as the side effects of the drug in the drug information leaflet the participants received as part of the placebo/nocebo induction.

## Method

### Participants

Seventy-five participants, 49 females (65.3%) and 26 males (34.7%), between 18 and 37 years old (*M* = 22.7, *SD* = 3.8) participated. Participants were recruited between March and June 2018 *via* e-mail advertisement [“Brain doping—Healthy participants wanted for an experiment on nootropics (smart drugs)”] addressed to staff and students of a German university. As cover story, participants were told that the goal of the study was to assess short-term effects of cognitive-performance-modulating drugs using a new delivery route (nasal spray). Participants were told that they would be randomly assigned to either a group receiving a fast acting stimulant (“Modafinil”) or a fast acting antiallergic agent (“Vividrin”) or no medication at all. In addition, they were informed that their cognitive performance would be tested using a computer-based cognitive performance task before and after drug administration. Actually, participants in the Modafinil and the Vividrin group both received the same placebo nasal spray without active ingredient. Inclusion criteria were age between 18 and 65 years, and fluency in German. Exclusion criteria were allergies to any substances actually (chili and sesame) or purportedly (Modafinil, Vividrin^®^) used in the study, pregnancy or nursing, suffering from a known mental disorder or severe medical condition, and intake of psychopharmacological drugs or prescription drugs used for enhancing cognitive performance within the last month before participation. All inclusion and exclusion criteria were assessed *via* self-report in a phone screening. Participants gave written informed consent and were paid 10€ for their participation. The experiment was conducted according to the Declaration of Helsinki and the local ethics committee approved the study protocol (#2018-0001).

The sample size was based on an *a priori* power analysis using G*Power 3 software (24) for our main outcome, the actual objective performance. For the 3 × 2 ANOVA interaction effect between three groups and two test of attentional performance (TAP) assessments, a total sample of at least 72 participants would be needed to detect a small effect (*f* = .15) with 95% power, alpha at .05, and correlation between repeated measurements (estimated on the basis of retest reliability described in the TAP manual) of.80.

The participants were randomly assigned in equal numbers to the placebo-Modafinil, nocebo-Vividrin^®^, and a natural history group. We observed no significant differences between the three groups regarding age, sex, and previous experience with performance-enhancing drugs (see **Table 1**). After completion of the experiment, seven participants (9%; placebo-Modafinil: *n* = 2, nocebo-Vividrin^®^: *n* = 3, natural history: *n* = 2) reported that they had not believed the cover story. Since the number of non-believers was similar across groups, these participants were not excluded from statistical analyses.

**Table 1:** Sample characteristics at baseline.

	Placebo-Modafinil (*n* = 25)	Nocebo-Vividrin^®^ (*n* = 25)	Natural history (*n* = 25)	Group effect
Age in years, M (SD)	22.5 (4.0)	22.7 (4.0)	22.8 (3.7)	*F*(2,72) = 0.043, *p* = .958
Number females, *n* (%)	13 (52.0%)	19 (76.0%)	17 (68.0%)	χ²(2) = 3.30, *p* = .192
Previous experience with performance modulation drugs, *n* (%)	0.0 (0.0%)	1.0 (4.0%)	1.0 (4.0%)	χ²(2) = 1.03, *p* = .598

### Questionnaires and Self-Ratings

#### Subjective Performance Expectation

**Figure 1:**
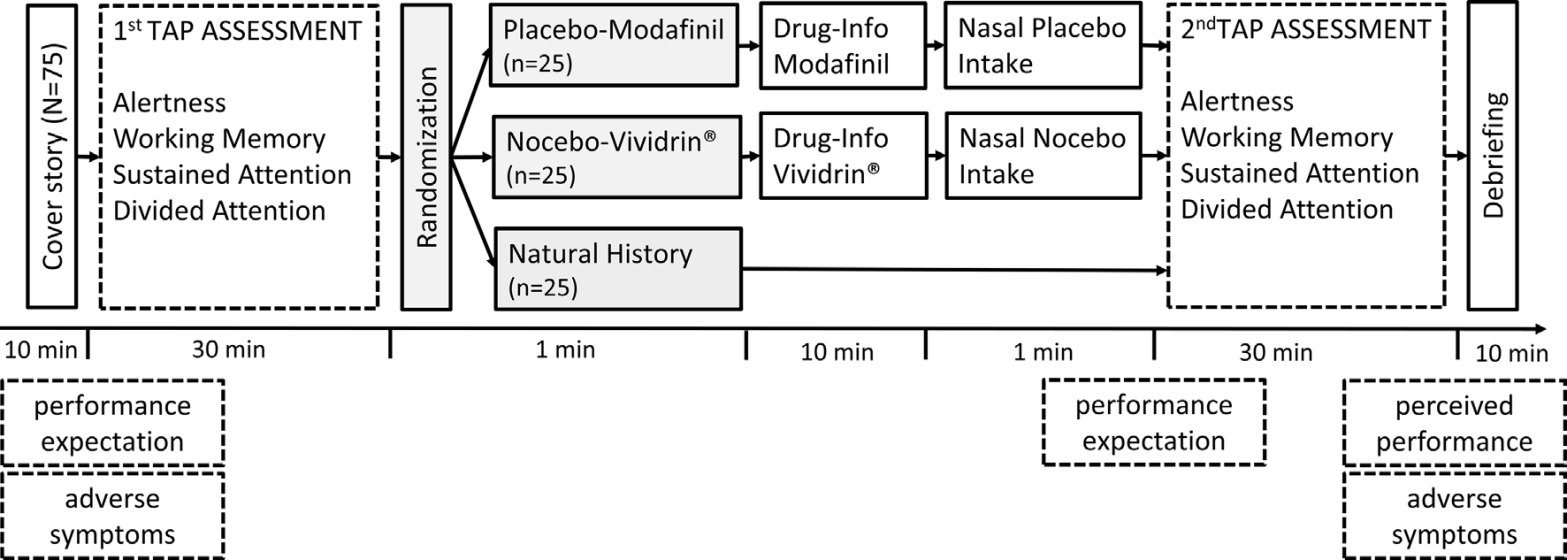
Study design (see Methods for details) and outcome measurements (dashed frames) for the placebo–Modafinil (*n* = 25), nocebo-Vividrin^®^ (*n* = 25), and natural history (*n* = 25) condition; TAP, Test of Attentional Performance.

To assess participant’s subjective performance expectation, we used the item “I will perform well in the task” to be rated on a seven-point Likert scale ranging from 1 (not agree at all) to 7 (totally agree). We assessed performance expectation online prior to each TAP assessment (see **Figure 1**).

#### Perceived Change in Cognitive Performance

Participants were asked to rate the perceived change in cognitive performance between the first and the second cognitive assessment (“How do you rate your cognitive performance now in comparison to the first assessment?”) on a visual analog scale (VAS) ranging from 1 (worse) to 100 (better). The rating was assessed online after the second TAP assessment.

#### Adverse Symptoms/“Side Effects”

Subjectively perceived adverse symptoms and side effects of the purportedly administered drugs were assessed using the Generic Assessment of Side Effects Scale (GASE) (25). The original GASE entails 36 symptoms and covers the most frequently reported side effects of medications in clinical trials. The severity of each symptom is rated on a four-point Likert scale ranging from “not present” (0) to “severe” (3). The GASE has good internal consistency (Cronbach’s α = 0.89) and has been validated (25). For the purpose of our study, 12 adverse symptoms were taken from the original GASE such that they matched the potential side effects as described in the drug information leaflet given to the participants (Modafinil: headache, palpitations/irregular heartbeat, abdominal pain, fatigue/tiredness and irritability/nervousness; Vividrin^®^: bitter taste, nausea, skin rash/itching, feeling of weakness and drowsiness/exhaustion; both drugs: dizziness, irritation of nose or throat). We selected those adverse symptoms that could be expected to occur relatively quickly following acute administration of the drug and to fluctuate over the course of the experiment. We followed the recommendation of Rheker et al. (26) and assessed adverse symptoms twice, before the first TAP assessment (as baseline) and after the second TAP assessment, since complaints about minor bodily symptoms are extremely common in the general population (base rates up to 80%) (27, 28) and might easily be misattributed to the nasal spray intake.

### Cognitive Performance

Cognitive performance was tested using the subtests Alertness, Working Memory, Sustained Attention, and Divided Attention of the computer-based TAP (29). The TAP is a well-established test battery for assessing various aspects of cognitive performance and is suitable for testing healthy subjects. For each TAP subtest, the test performance scores were determined according to the TAP manual (see **Table 2**). Alertness is tested by requiring participants to press a key as quickly as possible when they notice a cross on the monitor, which is displayed at randomly varying intervals (preceded or not preceded by a warning tone). Working Memory is tested by a modified N-1 back task, i.e., a sequence of numbers is presented on a computer screen, and participants are required to indicate whether or not the currently presented number matches the previously shown number or the one before. In the Sustained Attention test, a sequence of stimuli is presented on the monitor. Participants are required to press a key whenever the stimulus presented matches the preceding stimulus regarding one of two predetermined stimulus characteristics (color, shape, size, or filling). In the Divided Attention test, participants undergo a dual task, i.e., a visual (“press a key when a varying number of crosses on the monitor form a square”) and an auditory task (“press a key when a tone occurs twice in a row within a high and low tone sequence”). For all subtests that were used, the maximum level of difficulty was selected, whenever different levels of difficulty were available. As displayed in **Figure 1**, the TAP was assessed before and after manipulation of participants’ expectation.

**Table 2:** Cognitive performance (TAP scores at 1^st^ assessment and 2^nd^ assessment) per group and group*time interaction.

TAP Subtest	Placebo-Modafinil (*n* = 25)				Nocebo-Vividrin^®^ (*n* = 25)				Natural history (*n* = 25)				Group		Time		Group*time interaction	
	1st assessment		2nd assessment		1st assessment		2nd assessment		1st assessment		2nd assessment		*F*(2,72)	p	*F*(1,72)	p	*F*(2,72)	p
	M	SD	M	SD	M	SD	M	SD	M	SD	M	SD						
Alertness (without warning signal)																		
Median reaction time (ms)	235.3	33.7	237.7	34.5	231.2	27.0	235.3	31.4	241.0	49.5	240.0	45.8	0.27	.766	0.31	.578	0.21	.811
SD reaction time	32.9	17.5	44.2	27.7	34.6	22.0	39.4	22.9	33.8	16.2	41.6	23.9	0.04	.963	10.40	.002	0.57	.566
Alertness (with warning signal)																		
Median reaction time (ms)	232.6	33.7	231.1	37.5	226.7	20.8	227.2	24.7	236.7	39.3	232.0	44.5	0.33	.718	0.56	.458	0.35	.707
SD reaction time	29.7	13.6	35.6	20.9	32.9	14.1	36.5	20.0	33.6	15.6	36.8	23.6	0.16	.854	5.97	.017	0.24	.786
Number of lapses	1.24	0.72	1.16	0.62	1.16	0.69	1.08	0.81	1.24	0.93	1.20	0.50	0.27	.764	0.31	.577	0.01	.988
Number of Anticipations	0.44	0.71	1.28	1.59	0.76	1.05	1.32	1.75	0.56	1.08	1.52	2.02	0.18	.835	19.84	<.001	0.45	.639
Phasic Alertness (ms)	0.01	0.08	0.03	0.08	0.02	0.08	0.03	0.06	0.01	0.08	0.03	0.07	0.02	.981	3.18	.079	0.02	.978
Working Memory																		
Number of omissions	1.28	1.14	1.24	1.42	1.92	2.31	2.04	2.46	1.20	1.35	0.84	1.11	2.71	.073	0.25	.620	0.57	.569
Number of errors	1.36	1.38	0.80	1.08	0.88	1.01	0.76	0.93	1.20	1.19	0.48	0.65	0.74	.482	9.65	.003	1.43	.247
Sustained Attention																		
Number of omissions	7.60	7.11	8.36	7.45	7.60	5.69	9.16	7.42	7.88	5.88	8.04	6.77	0.03	.966	2.75	.101	0.66	.519
Number of errors	5.28	5.73	2.80	4.35	7.60	8.52	3.68	5.23	7.36	12.63	4.76	10.40	0.45	.642	27.12	<.001	0.64	.530
Divided Attention																		
Number of omissions	1.08	1.47	1.64	1.91	1.40	2.61	1.88	2.82	1.12	1.17	1.44	1.83	0.28	.755	3.65	.060	0.09	.915
Number of errors	0.96	1.40	0.84	1.86	1.04	1.37	0.92	1.12	0.80	0.91	0.52	0.71	0.56	.574	1.37	.245	0.13	.878

TAP, Test of Attentional Performance.

### Experimental Setup

In the current randomized controlled parallel group design study, the primary outcome was actual cognitive performance analyzed *via* a 2 × 3 mixed model ANOVA with the repeated factor time (first TAP assessment vs. second TAP assessment) and the between group factor group (placebo vs. nocebo vs. natural history). Secondary outcomes were performance expectation, perceived performance, and adverse symptoms. All participants underwent the first cognitive test battery (TAP) as baseline measurement (see **Figure 1**). Then, participants were randomly assigned to one of three groups (placebo-Modafinil, nocebo-Vividrin^®^, natural history). Participants allocated to the placebo-Modafinil group were informed that they will receive a stimulating drug that enhances cognitive performance and increases general alertness. Participants allocated to the nocebo-Vividrin^®^ group were informed that they will receive a drug that dampens the activity of the central nervous systems and reduces alertness. Both groups actually received an active placebo nasal spray consisting of a mixture of sesame oil and capsaicin (0.0007%). Participants in the natural history group did not receive the nasal spray and were not further instructed regarding (potential) drug administration. Investigators were partially blinded to group allocation, since the participant leaflet for Modafinil or Vividrin^®^ was handed to the participants in a closed envelope. Hence, the experimenter was unaware of whether the participant received the Modafinil or the Vividrin^®^ instruction together with the nasal spray. For participants allocated to the natural history group, the experimenter was unblinded, since the participants were instructed to inform the experimenter that they were not supposed to take any nasal spray after reading the leaflet. After the information about the purported drug was given, participants in the placebo-Modafinil and nocebo-Vividrin^®^ group received the active placebo nasal spray. Participants were instructed to wait for 60 s after the drug application in order to ensure good absorption before undergoing the second performance test. Subjective performance expectation was measured prior to each TAP assessment. The perceived change in performance was rated after the second TAP test. Adverse symptoms were assessed before the first TAP test and after the second TAP test. Participants assigned to the natural history group underwent the same procedure; however, they received no nasal spray (see **Figure 1**).

### Study Procedure

Individuals interested in the study underwent a telephone screening to examine inclusion and exclusion criteria and to arrange a lab appointment. The participants were seated in a lab with the experimenter running the experiment from an adjacent room. The participants were monitored using a camera; they could communicate with the experimenter using a microphone at any time. After giving informed consent, participants completed the questionnaires online. Then they underwent the experiment (for details, see the section Experimental Setup). After completing the experiment, participants were asked to indicate whether or not they had believed the cover story, and they were then debriefed following a standardized protocol and were paid. The experiment lasted about 90 min in total.

### Statistical Analysis

Statistical analyses were performed using IBM SPSS Statistics 23.0 for Windows (Chicago, SPSS, Inc.). Group differences in age, sex and previous experience with performance-enhancing drugs at baseline were analyzed using univariate analysis of variance (ANOVA) and chi-square tests.

Group differences regarding change in performance expectation over time were tested using a mixed design ANOVA with *time* (before and after expectation manipulation) as within subject and *group* (placebo-Modafinil, nocebo-Vividrin^®^, natural history) as between group factors. Significant group × time interaction effect was followed up by Bonferroni-corrected *post hoc* tests, mean differences (*M* *_diff_*) are reported.

Group differences in perceived change of cognitive performance were tested using a univariate ANOVA, followed by Bonferroni-corrected *post hoc* tests; mean differences (*M* *_diff_*) are reported.

To test for group differences in change of actual cognitive performance, we carried out mixed design ANOVAs with *time* (first and second TAP assessment) as repeated measures and *group* (placebo-Modafinil, nocebo-Vividrin^®^, natural history) as between-group factor for each TAP subtest performance score as dependent variable.

Group differences in drug-specific and unspecific adverse symptoms (“side effects”) as described in the drug information leaflet assessed following the second TAP assessment were evaluated in an exploratory analysis using ANCOVAs for each item with symptom intensity prior to the first TAP assessment used as covariate, respectively. For this exploratory analysis, the family-wise error rate was set at .10. Bonferroni correction led to a *p*-value of .02 for single comparisons with respect to drug specific symptoms, and .05 as criterion for significance for single comparisons with respect to unspecific symptoms. Significant ANCOVAs were followed up by Bonferroni-corrected pairwise *post hoc* comparisons. Mean differences (*M*_diff_) are reported.

Product-moment correlation analyses were conducted to test the relationship between change in performance expectation and perceived change in performance.

## Results

### Performance Expectations and Actual Cognitive Performance

#### Performance Expectation

**Figure 2:**
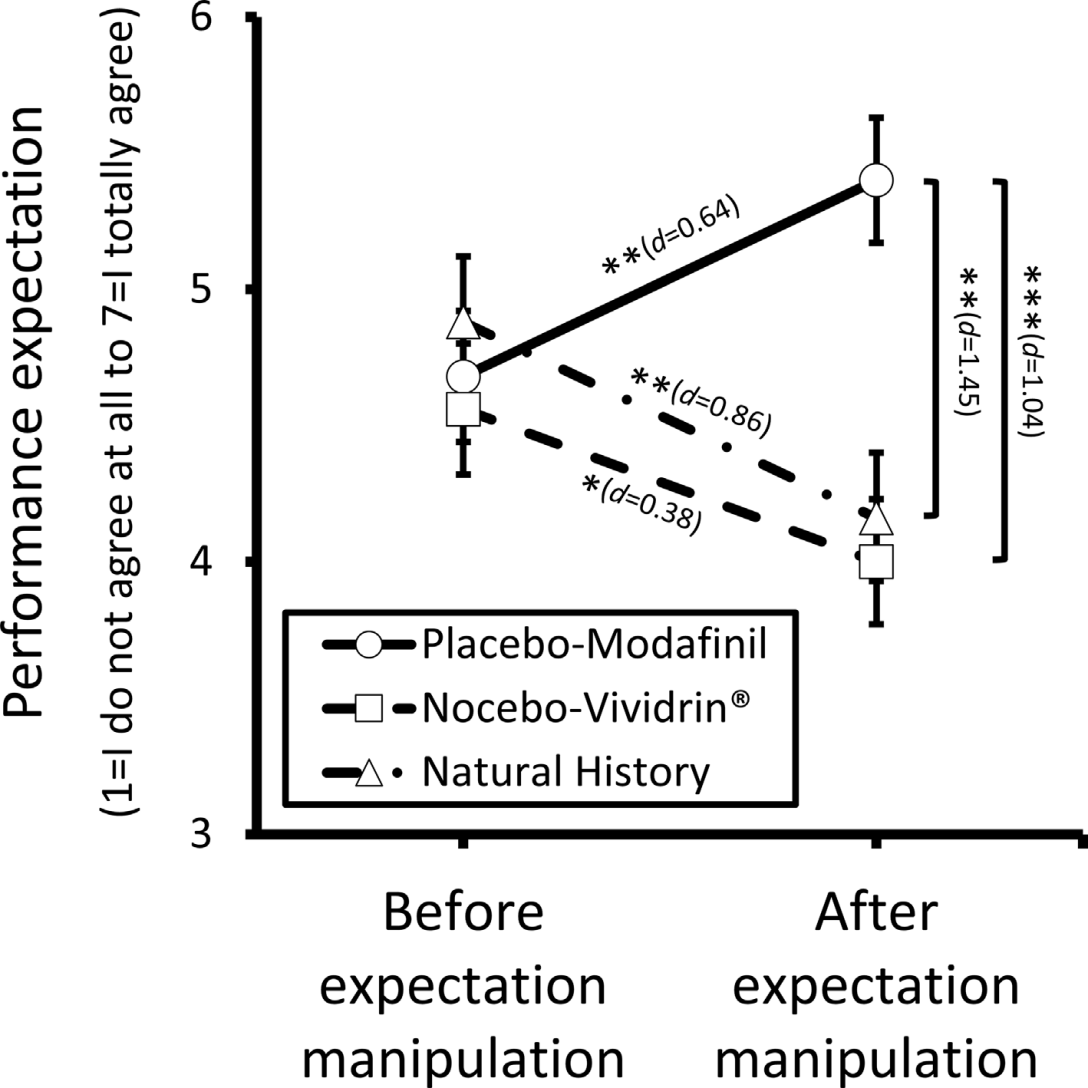
Performance expectation (“I will perform well in the task”) before the first TAP assessment and after expectation manipulation *via* drug information and nasal placebo intake. Error bars represent standard errors. *d* = Cohen’s *d*. *p < .05. **p < .01. ***p < .001.

The mixed-measure ANOVA revealed a significant interaction effect between group and time [*F*(2,72) = 8.74, *p* < .001], a main effect of group [*F*(2, 72) = 4.01, *p* = .022], but no significant main effect of time. Follow-up tests revealed that the groups differed significantly in their performance expectation after expectation manipulation, but not at baseline (see **Figure 2**). Participants in the placebo-Modafinil group (*M* = 5.4, *SD* = 0.23) endorsed a significantly higher performance expectation than participants in the nocebo-Vividrin^®^ (*M* = 4.0, *SD* = 0.23, *M*_diff_ = 1.4, *p* < .001, *d* = 1.04) and in the natural history group (*M* = 4.2, *SD* = 0.23, *M*_diff_ = 1.2, *p* = .001, *d* = 1.45), after expectation manipulation. There was no significant difference in performance expectation between the nocebo-Vividrin^®^ and the natural history group (*M*_diff_ = 0.2, *p* = 1.000). Moreover, performance expectation increased significantly in the placebo-Modafinil group following the expectation manipulation (*M*_diff_ = 0.72, *p* = .009, *d* = 0.64), and it decreased significantly in the nocebo-Vividrin^®^ group (*M*_diff_ = −0.56, *p* = .039, *d* = −0.38) and the natural history group (*M*_diff_ = −0.72, *p* = .009, *d* = −0.86).

#### Perceived Change in Performance Between First and Second Assessment TAP

**Figure 3:**
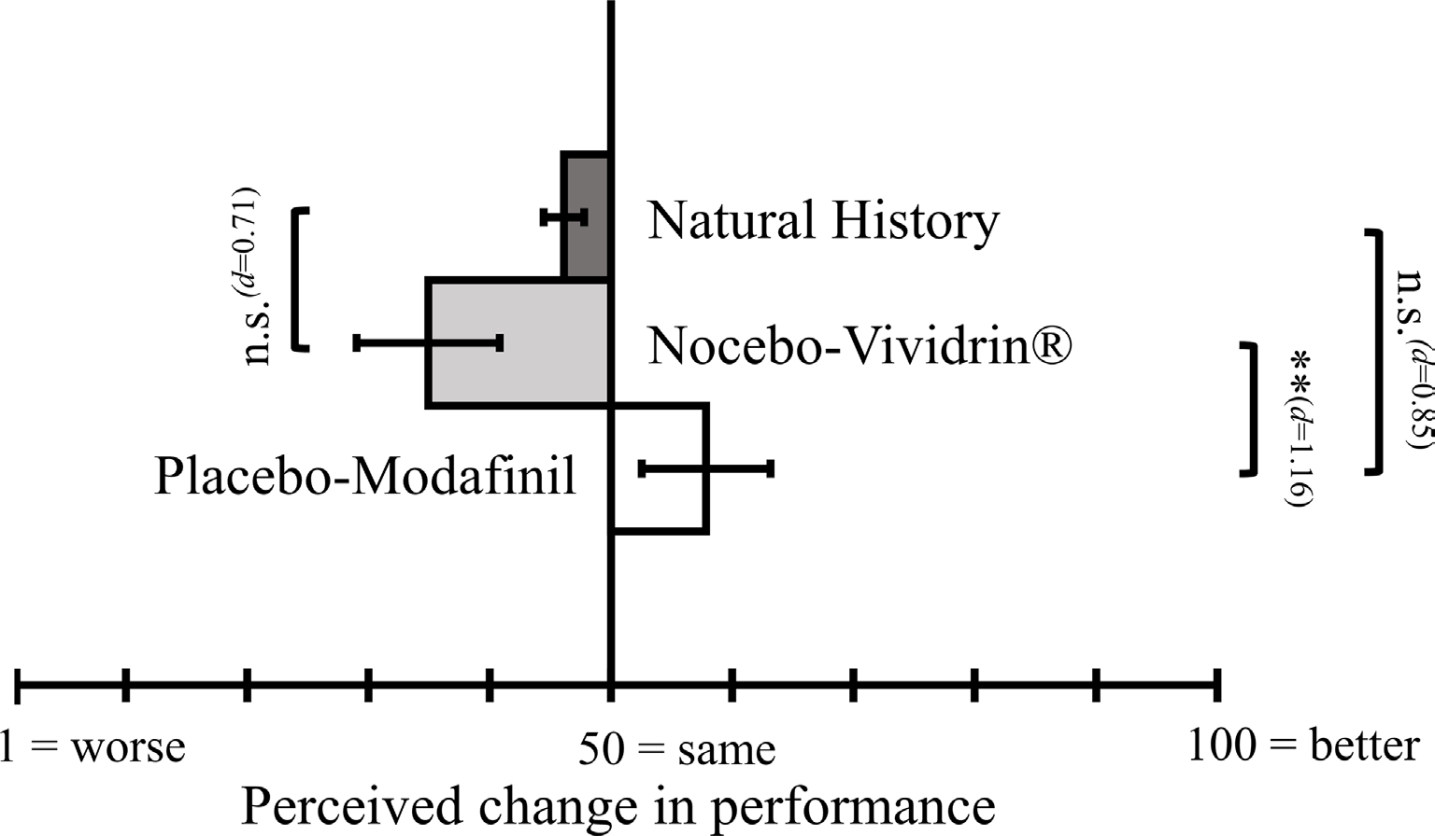
Perceived change in performance (“How do you rate your cognitive performance now in comparison to the first assessment?”) using a 1–100 VAS (1 = “worse”; 100 = “better”). Error bars represent standard errors. **p < .01.

The univariate ANOVA yielded a significant group main effect [*F*(2,38) = 6.37, *p* = .004]. As illustrated in **Figure 3**, Bonferroni-corrected *post hoc* tests revealed that the placebo-Modafinil group reported significantly greater improvement in performance than the nocebo-Vividrin^®^ group (*M*_diff_ = 22.91, *p* = .003, *d* = 1.16). Neither the placebo-Modafinil group (*M*_diff_ = 11.71, *p* = .190, *d* = 0.85) nor the nocebo-Vividrin^®^ group (*M*_diff_ = −11.20, *p* = .207, *d* = 0.71) differed from the natural history group with respect to perceived change in performance.

#### Relationship Between Performance Expectation and Perceived Change in Performance

In the placebo-Modafinil and the nocebo-Vividrin^®^ group combined, there was a significant positive correlation between performance expectation after placebo intake and perceived change in performance from the first assessment TAP to the second assessment TAP (*r* = .47, *p* = .002) as measured after the second assessment TAP.

#### Actual Cognitive Performance

There was no statistically significant interaction effect between group and time (first assessment vs. second assessment) for any of the TAP performance indices, as displayed in **Table 2**. Thus, there was no evidence for a differential effect of group allocation on actual cognitive performance. Moreover, no significant group main effects emerged. However, there were significant main effects for time, with respect to some subtests. Alertness without warning signal standard deviation reaction time [*F*(1, 72) = 10.40, *p* = .002, partial η² = .126], Alertness with warning signal standard deviation reaction time [*F*(1, 72) = 5.97, *p* = .017, partial η² = .077], and Alertness with warning signal number of anticipations [*F*(1, 72) = 19.84, *p* < .001, partial η² = .216] show higher values at the second assessment, respectively. Working Memory number of errors [*F*(1, 72) = 9.65, *p* = .003, partial η² = .118] and Sustained Attention number of errors [*F*(1, 72) = 27.12, *p* < .001, partial η² = .274] show lower values at the second assessment, respectively.

### Adverse Symptoms (“Side Effects”)

There was a significant difference in fatigue [*F*(2,71) = 4.41, *p* = .016] and irritation of nose or throat [*F*(2,71) = 29.82, *p* < .001] between groups after the second TAP assessment as revealed by ANCOVAs with symptom intensity prior to the first TAP assessment as covariate (see **Table 3**).

**Table 3:** Intensity of the 12 selected GASE adverse symptom items before 1^st^ TAP assessment (baseline) and post 2^nd^ TAP assessment.

	Placebo-Modafinil (*n* = 25)		Nocebo-Vividrin^®^ (*n* = 25)		Natural history (*n* = 25)		Difference between groups post 2^nd^ assessment (with pre 1^st^ assessment as covariate)	
	Pre 1^st^ assessment, M (SD)	Post 2^nd^ assessment, M (SD)	Pre 1st assessment, M (SD)	Post 2nd assessment, M (SD)	Pre 1st assessment, M (SD)	Post 2nd assessment, M (SD)	*F*(2,71)	p
Placebo-Modafinil specific								
Headache	0.20 (0.41)	0.36 (0.57)	0.28 (0.46)	0.56 (0.65)	0.28 (0.54)	0.52 (0.65)	0.49	.616
Palpitations, irregular heartbeat	0.00 (0.00)	0.16 (0.62)	0.12 (0.44)	0.08 (0.28)	0.12 (0.33)	0.00 (0.00)	1.44	.243
Abdominal pain	0.04 (0.20)	0.00 (0.00)	0.20 (0.65)	0.28 (0.68)	0.04 (0.20)	0.04 (0.20)	2.45	.094
**Fatigue (tiredness)**	**0.64 (0.64)**	**0.92 (0.91)**	**0.92 (0.70)**	**1.76 (0.83)**	**1.00 (0.76)**	**1.44 (0.96)**	4.41*	**.016**
Irritability, nervousness	0.24 (0.44)	0.16 (0.62)	0.32 (0.48)	0.20 (0.50)	0.40 (0.58)	0.08 (0.28)	0.83	.441
Nocebo-Vividrin^®^ specific								
Bitter taste	0.12 (0.33)	0.12 (0.33)	0.00 (0.00)	0.32 (0.56)	0.04 (0.20)	0.08 (0.28)	2.67	.076
Nausea	0.00 (0.00)	0.00 (0.00)	0.00 (0.00)	0.08 (0.28)	0.00 (0.00)	0.04 (0.20)	1.03	.363
Skin rash or itching	0.08 (0.28)	0.04 (0.20)	0.12 (0.33)	0.08 (0.28)	0.12 (0.44)	0.12 (0.44)	0.75	.477
Feeling of weakness	0.08 (0.28)	0.12 (0.33)	0.12 (0.33)	0.44 (0.77)	0.20 (0.50)	0.28 (0.46)	2.14	.126
Drowsiness (exhaustion)	0.52 (0.59)	0.84 (0.94)	0.76 (0.66)	1.48 (0.77)	0.64 (0.64)	1.32 (0.85)	2.87	.063
Unspecific								
Dizziness	0.04 (0.20)	0.40 (0.71)	0.04 (0.20)	0.32 (0.56)	0.12 (0.33)	0.28 (0.46)	0.65	.525
**Irritation of nose or throat**	**0.16 (0.47)**	**1.00 (0.58)**	**0.24 (0.44)**	**1.04 (0.54)**	**0.08 (0.28)**	**0.08 (0.28)**	29.82***	**<.001**

GASE, Generic Assessment of Side Effects Scale, Bonferroni correction of the family wise error rate led to a p-value of .02 as criterion for significance with respect to the drug specific symptoms and .05 as criterion for significance with respect to unspecific symptoms.

*p < .02, ***p < .001.

*Fatigue.* In comparison to participants in the nocebo-Vividrin^®^ group, participants in the placebo-Modafinil group reported significantly less fatigue after placebo treatment (*M*_diff_ = 0.84, *p* = .005, *d* = 0.96). However, there was no significant difference between the natural history group and the placebo-Modafinil group (*M*_diff_ = 0.52, *p* = .136, *d* = 0.56) or nocebo-Vividrin^®^ group (*M*_diff_ = 0.32, *p* = .641, *d* = 0.36), with respect to fatigue post second TAP assessment.

*Irritation of nose and throat*. In comparison to participants in the natural history group, participants in the placebo-Modafinil group (*M*_diff_ = 0.92, *p* < .001, *d* = 2.02) and the nocebo-Vividrin^®^ group (*M*_diff_ = 0.96, *p* < .001, *d* = 2.23) reported significantly more irritation of their nose and throat after the placebo intervention.

## Discussion

Our key finding is that manipulation of performance expectation *via* a placebo cognitive performance enhancing nasal spray affects the perceived change in performance and tiredness, but not the actual cognitive performance in healthy adults. Reasons for nonmedical use of prescription stimulants among university students are to improve concentration, to perform better in university (2), to “catch up with high achieving students,” to increase the amount of work done under time constraint, to improve energy, and to “pull an all-nighter” (30). Therefore, the demonstrated placebo effect affecting subjective outcomes like perceived performance and tiredness could partially explain why these drugs are used despite potential risks and unclear benefit.

As hypothesized, the placebo-Modafinil group showed a significantly higher performance expectation after the expectation manipulation than the nocebo-Vividrin^®^ and the natural history group. Although nearly all prior studies assumed that *a priori* performance expectation was changed by the intervention (e.g., administration of a placebo pill or verbal suggestion), the majority of these studies did not assess performance expectation directly after the intended expectation manipulation. Rather, the change in performance expectation was extrapolated based on a *post hoc* performance rating (9, 10, 14, 16–21). Clearly, *a priori* performance expectations and *a posteriori* performance ratings tap different aspects. Indeed, we observed only a moderate positive correlation between performance expectation after placebo intake and perceived change in performance (*r* = .47). Moreover, the few studies that directly assessed *a priori* performance expectation (13, 15) failed to report changes in performance expectation due to their intervention. Hence, it is unclear whether the intervention actually resulted in change in expectation. In the present study, we carefully assessed performance expectation prior and subsequent to the placebo instruction and observed a medium-sized (*d* = 0.64) increase in performance expectation within the placebo-Modafinil group.

Contrary to our hypothesis that a positive performance expectation would improve actual performance, there were no group differences in actual cognitive performance. This finding is consistent with the study of Looby and Earleywine (13), but inconsistent with the finding of an improvement in sustained attention in participants believing that they had received a cognitive-enhancing drug (16). Interestingly, in the latter study, performance expectation was induced by providing false feedback that participants had improved their performance by 20% due to the pill they had taken before in a blinded manner. Hence, based on their apparent change in performance, participants formed their belief about whether or not they had taken active pill or the placebo. As is long known, (perceived) mastery of a task has a strong effect on self-efficacy (31). In a similar vein, beliefs based on (seeming) changes in performance are likely to be more credible and powerful than verbal suggestion for the participants. This is also consistent with findings that verbal suggestion as compared to conditioning is associated with a smaller placebo effect (32). Oken et al. (15) also found an improvement of actual cognitive performance (memory and attention) after placebo pill intake. This may be explainable by the fact that Oken et al. (15) investigated performance-enhancing placebo effects in a sample of healthy seniors, 65–85 years of age. In elderly individuals, a placebo effect might manifest itself more easily because any ceiling effect is unlikely due to lower baseline levels of cognitive functions such as attention or memory. In line with such an interpretation, Oken et al. (15) reported that even in their sample of elderly, older participants demonstrated a greater benefit from placebo intake. Indeed, Oken et al. (15) relied on a neuropsychological assessment battery typically used for dementia screening (CERAD), whereas the TAP used in the current study is also sensitive for measuring high levels of cognitive functioning. Moreover, given the role of medication-related beliefs (33), a potential confounding influence could be that the attitude towards neuroenhancement as treatment for a cognitive deficit in elderly is quite different than the expected effects of drugs used for “brain doping” by healthy young adults.

As predicted, participants in the placebo-Modafinil group rated their perceived change in cognitive performance subsequent to the application of the nasal spray significantly better (*d* = 1.16) compared to the nocebo-Vividrin^®^ group. Hence, an enhanced performance expectation affects the perceived change in performance, irrespective of any changes in actual cognitive performance. Similar observations, i.e., that performance expectation affects the perceived change in performance, but not the actual cognitive performance, have been made previously [e.g., Ref. (18)]. As outlined by Schwarz and Büchel (18), it is possible that objective measures of cognitive performance are generally not susceptible to expectancy manipulation. Those studies demonstrating an expectancy-induced change in objective performance (15, 16) are at odds with such an assumption. Alternatively, it is possible that only specific cognitive functions are susceptible to expectancy manipulation (e.g., tasks entailing a motivational component and/or tasks requiring great effort). Previous studies vary considerably with regard to the specific type of cognitive task used to evaluate changes in performance. For example, implicit learning task (19) or tests of fluid intelligence (21) have been used. Taking into account previous reports on changes in cognitive functioning due to administration of cognitive enhancers in healthy participants, we decided to focus on attention as a core cognitive function rather than complex cognitive functions (e.g., problem solving). We choose the TAP due to the broad range of functioning it allows to test. However, the TAP was developed to allow a differential diagnosis of attention deficits, based on reference data in the general population. Clearly, in our sample, there is no evidence for a potential ceiling effect. The mean T-values range between 46 and 57 for the different performance indices, indicating average cognitive performance in our healthy sample. At this point, it is far from being clear which method for expectation manipulation, e.g. sham subliminal presentation of information (20) or smelling an odor (19) or verbal suggestion, is particularly effective in yielding actual changes in performance. Moreover, it is unclear which aspects of cognitive functioning are susceptible to a placebo manipulation. Finally yet importantly, design differences (e.g., balanced placebo design vs. between group designs) could account for the heterogeneous results.

Contrary to our hypotheses, there was no difference in performance expectation between the nocebo-Vividrin^®^ and the natural history group, indicating that the intended manipulation of the performance expectation had failed for the nocebo-Vividrin^®^ group. Our results show that both groups, the nocebo-Vividrin^®^ group (*d* = −0.38) and the natural history group (*d* = −0.86), showed a significant decrease in performance expectation compared to baseline with the effect sizes suggesting a larger drop. Possibly, participants in the natural history group, who were interested in participating in a study on brain doping as advertised, were disappointed that they were assigned to the control (natural history) group and therefore did not have the chance to try a smart drug, thus resulting in a nocebo effect. Participants in the nocebo-Vividrin^®^ group may have underestimated the effects of Vividrin^®^ due to its being administered as part of study on brain doping. Alternatively, participants may have had prior experiences with Vividrin^®^, which is a common anti-allergic substance, and, based on their own experience, did therefore not expect a deteriorated cognitive performance. As described above, the majority of studies did not directly assess performance expectation; hence, there are no previous findings on whether performance expectation is susceptible to negative manipulation in the same way as it is to positive manipulation.

We also attempted to evoke adverse symptoms consistent with the side effect profiles of the placebo/nocebo medications as listed in the drug information leaflets given to the participants. There was no evidence for a drug-specific side effect profile in either experimental group. Yet, the description of adverse symptoms is known to influence participants’ perception of bodily symptoms (34). Fatigue was described as a potential side effect of Modafinil. Interestingly, participants in the placebo-Modafinil group felt less tired after the second TAP test than the nocebo-Vividrin^®^ group. This suggests that describing Modafinil as a stimulating drug, which facilitates general alertness, overshadowed the listed side effects, especially given that fatigue as a side effect might seem counterintuitive for a stimulating drug. Moreover, since increased alertness and prolonged endurance when working are known reasons for nonmedical use of prescription stimulants (30), disregarding tiredness as an unwanted side effect could partly explain why these drugs are used despite potential risks and unclear benefit, especially if the effect could be evoked even by a medication without active component (a placebo).

There were no group differences regarding the other complaints, except for irritation of nose and throat, which is attributable to the capsaicin in the nasal spray, and therefore was reported significantly more often in the experimental groups as compared to the natural history group. Possibly, participants assumed that a single dose of the study medication would not lead to the side effects as described, but, based on previous experiences when taking medications, implicitly assumed that such side effect would primarily occur when regularly taking the same medication.

### Limitations

First of all, we cannot rule out a certain self-selection bias of the participants such that we may have tested primarily individuals willing to try a cognitive-performance-modulating drug using a new route of delivery (see the cover story of the study) in an experimental setting and/or individuals with prior experience with such drugs. Additionally, due to the cover story, participants might have expected to get the chance of trying a performance-enhancing drug and were disappointed when they were allocated in the nocebo or natural history group, potentially leading to a reduced motivation and commitment.

Furthermore, our findings are limited to healthy adults. As stated by Fuhr and Werle (17), psychological interventions for enhancing cognitive performance might even be more effective for patients with impaired cognitive functioning, e.g., when suffering from an affective disorder. Patients might be more susceptible to expectancy manipulation and might benefit both subjectively and objectively, for example, due to better concentration, greater motivation, and higher perceived self-efficacy.

The approach to evoke adverse symptoms *via* information provided in the drug information leaflets may have not been optimal as they were described next to the drug action effects. Participants may have focused on the potentially desired effects and may have disregarded the adverse effects, especially if assuming, based on personal experience that “side effects” occur primarily when a drug is taken repeatedly.

We also cannot rule out that the placebo and the nocebo instruction might have been not fully equivalent since we referred to the substance name in the placebo condition (Modafinil), but used the trade name in the nocebo condition (Vividrin^®^). Given that Vividrin^®^ is relatively well known, this might have triggered more expectations, thus confounding our expectancy manipulation.

In placebo/nocebo studies, in general, the situational context strongly influences study outcomes. Participants may have not fully believed that an actual drug, especially with negative effects on cognitive performance, would be applied at a department of psychology, especially with no physician being ostensibly involved.

Finally, it should be noted that attentional performance is just one facet of cognitive performance. However, unlike most previous studies, we used several tests of attentional performance rather than relying on just one or two tests. If performance expectancy primarily alters those cognitive functions entailing for example a strong motivational component, future studies should seek to use more comprehensive cognitive test batteries to elucidate which cognitive functions may be susceptible to performance expectancy effects.

To our knowledge, the present study is the first study investigating expectancy effects in pharmacological neuroenhancement including both placebo and nocebo instructions, assessing performance expectation directly after the intended manipulation and perceived change in cognitive performance, as well as cognitive measures. Additionally, it is the first study investigating drug-specific side effects of placebo- and nocebo-medication in the context of pharmacological neuroenhancement.

### Conclusions

Manipulation of performance expectation affects the perceived change in performance and tiredness in healthy adults. This may explain why college students use such drugs despite their small, if any impact on actual cognitive functioning. Therefore, future studies should systematically assess the role of performance expectation, perceived change in performance, and tiredness in predicting future use of prescription drugs to improve cognitive performance. Future studies should also address whether enhancing placebo effects could be helpful in improving perceived or actual deficits in cognitive performance. This could stimulate clinical studies on utilizing placebo effects in clinical practice, for example, in patients suffering from affective disorders. Future studies should entail different cognitive tasks such that it can be determined what makes a cognitive task susceptible to expectancy manipulation. This holds the opportunity to elucidate the underlying mechanisms of such placebo/nocebo responses. With respect to the effect of nocebo responses on cognitive performance, our results suggest that demonstrating differences between a nocebo group (expectation manipulation intended to decrease performance expectation) and a natural history group seems to be challenging, due to potential nocebo effects within the natural history group. Nevertheless, in direct comparison with a placebo group (expectation manipulation intended to increase performance expectation) our data give evidence that a nocebo-intervention affects the perceived change in performance, irrespective of any changes in the actual cognitive performance. Future studies should apply alternative approaches to a natural history control group. Additionally maybe it would be beneficial to separate studies addressing placebo and nocebo effects in cognitive performance to avoid expectation violation of participants interested to try a pharmacological neuroenhancer and receiving no medication at all or a substance supposed to provoke the opposite effect.

The present findings add to the growing body of evidence that highlights the influence of prescription-stimulant-related expectancies on subjective outcomes but not cognitive performance. This finding implies that more information about the role of subjective expectations and the discrepancy between subjectively perceived and actual changes in cognitive performance needs to be communicated to the public in an attempt to modify beliefs held by (potential) users, thus possibly correcting individual beliefs about the benefit of such drugs.

## Data Availability

The datasets generated for this study are available on request to the corresponding author.

## Ethics Statement

This study was carried out in accordance with the recommendations of the local ethics committee of the faculty of psychology at Justus-Liebig-University Giessen, Germany with written informed consent from all subjects. All subjects gave written informed consent in accordance with the Declaration of Helsinki. The protocol was approved by the local ethics committee of the faculty of psychology at Justus-Liebig-University Giessen, Germany.

## Author Contributions

Both authors contributed to the conception and design of the study. AW and CH conducted the statistical analysis. AW wrote the first draft of the manuscript. Both authors approved the submitted version.

## Conflict of Interest Statement

The authors declare that the research was conducted in the absence of any commercial or financial relationships that could be construed as a potential conflict of interest.
